# The prostaglandin synthases, COX-2 and L-PGDS, mediate prostate hyperplasia induced by low-dose bisphenol A

**DOI:** 10.1038/s41598-020-69809-y

**Published:** 2020-08-04

**Authors:** Shuangshuang Wu, Dongyan Huang, Xin Su, Han Yan, Aicui Ma, Lei Li, Jianhui Wu, Zuyue Sun

**Affiliations:** 1 Pharmacy School of Fudan University, Shanghai, 201203 China; 2 National Evaluation Centre for Toxicology of Fertility Regulating Drugs, Shanghai Institute of Planned Parenthood Research, Shanghai, 200032 China; 3 Key Laboratory of Reproduction Regulation of NPFPC, Shanghai, 200032 China; 4 Reproductive and Developmental Research Institute of Fudan University, Shanghai, 200032 China

**Keywords:** Toxicology, Molecular biology

## Abstract

This study aimed to identify prostaglandin synthases (PGS) that mediate bisphenol A (BPA)-induced prostatic hyperplasia and explore their underlying mechanisms. In an in vivo study, male adult Sprague–Dawley rats were treated with different concentrations of BPA (10, 30, 90, or 270 μg/kg, i.g., daily), or with vehicle for 4 weeks. Results revealed that low-dose BPA induced prostatic hyperplasia with increased PCNA/TUNEL ratio. It significantly upregulated the expression of cyclooxygenase-2 (COX-2) and NF-κB in the dorsolateral prostate (*P* < 0.05) and the expression of lipocalin-type prostaglandin D synthase (L-PGDS) in ventral prostate (*P* < 0.05). The level of estradiol (E_2_)/testosterone (T) and expression of androgen receptor (AR) and estrogen receptor α (ERα) were also altered. In vitro studies showed that low-dose BPA (0.1–10 nM) promoted the proliferation of human prostate fibroblasts and epithelial cells, and significantly upregulated the expression of COX-2 and L-PGDS in the cells. The two types of cell proliferation induced by BPA were inhibited by COX-2 inhibitor (NS398) and L-PGDS inhibitor (AT56), with increased apoptosis level. These findings suggested that COX-2 and L-PGDS could mediate low-dose BPA-induced prostatic hyperplasia through pathways involved in cell proliferation and apoptosis, which might be related to the functions of ERα and AR. The role of COX-2/NF-κB pathway in dorsolateral prostate requires further research.

## Introduction

Bisphenol A (BPA) is a synthetic plasticizer that is widely used to package daily necessities^1^. Environmental exposure to BPA has potential toxicity to the tissues of male system including those of the testes and prostate^2^, leading to abnormal prostate development and a trend of hyperplasia^3^. Prostate epithelial cells and prostate fibroblasts are the two main cell that constitute prostate tissues. Excessive proliferation of epithelial cells and prostate fibroblasts, and the transformation of epithelial cells to mesenchymal cells are involved in the pathogenesis of prostate hyperplasia^4,5^. Low-dose BPA (0.01-1 nM) has been reported to promote the proliferation of primary prostate epithelial cells in rats^6^. Similar to estradiol (E_2_), BPA promotes the proliferation of human prostate epithelial stem cells and increases the possibility of human prostate epithelial carcinoma^7^. However, the underlying mechanism of its effect on prostate cell proliferation and prostate hyperplasia remains unclear. As a typical environmental endocrine disruptor (EDC), BPA can disturb the endocrine functions by mimicking, enhancing, or inhibiting the endogenous estrogen activity, interfering with the androgen system^8^, and affecting the expressions of endocrine hormone-related genes and pathways.

Prostaglandin synthases (PGS) catalyze the formation of different active prostaglandins (PGs) in the arachidonic acid metabolic pathway. There are four main PGS that are closely associated with hormonal function. Cyclooxygenase-2 (COX-2) is an inducible prostaglandin H synthase that is less expressed in normal tissues but highly expressed when induced by cell-growth factors, inflammatory factors, and hormones^9^. In the male reproductive system, the overexpression of COX-2 is related to testosterone-induced hyperplasia, proliferation of seminal vesicle cells, and prostate cancer invasion^10^. COX-2/PGE signaling pathway is involved in the progression of benign prostatic hyperplasia (BPH)^11^. PGE_2_ synthase (PGES) catalyzes the conversion of PGH_2_ into prostaglandin E_2_. Similar to COX-2, the membrane-bound prostaglandin E_2_ synthase1 (mPGES-1) is generally overexpressed in hormone-sensitive diseases. In addition, mPGES-1/PGE_2_ mediates the development and progression of prostate cancer^12,13^. As a responsive inflammatory factor, NFκB interacts with the COX-2/PGE pathway to mediate the progressions of prostatitis^14^ and prostate cancer^15^. Lipocalin-type prostaglandin D synthase (L-PGDS) is a bifunctional protein that catalyzes the synthesis of prostaglandin D_2_ and functions as a transporter of lipophilic substances. The production and action of L-PGDS are regulated by the androgen hormone in the male reproductive system. Testosterone and testicular secretion assist in restoration and increase of the L-PGDS level in rat epididymis after castration^16^. Furthermore, L-PGDS/PGD_2_ mediates androgen-induced male alopecia (AGA)^17^, which is subsequently associated with a high incidence of BPH^18^. Our previous study found that after BPA administration, the PGDS transcription is increased, which might lead to the occurrence of prostate hyperplasia^19^. Prostaglandin F synthase (PGFS) is a terminal enzyme that catalyzes PGH_2_ and PGD into prostaglandin F. As a type of PGFS, Aldosterone reductase (AKR1C3) interconverts testosterone with delta(4)-androstene-3,17-dione, inactivates 5alpha-DHT to reduce active androgens in the prostate, and converts delta(4)-androstene-3,17-dione to testosterone (a substrate aromatizable to 17 beta-oestradiol) to enhance estrogenic activity in the mammary gland^20^. Thus, AKR1C3 is regarded as a target of different hormone-dependent diseases including prostate cancer^21^.

Our previous study revealed that BPA upregulated the transcriptional level of prostaglandin D_2_ synthase (PTGDS) gene, inducing prostate hyperplasia in adult rats^19^. PGS might be involved in the development of prostate, and is closely associated with the progression of prostate diseases. As BPA can interfere with the action of hormones and affect the normal development of prostate during the interaction of PGS with endocrine hormones, we hypothesized that PGS is involved in BPA-induced prostatic hyperplasia.

## Materials and methods

### Animal treatment

Male Sprague–Dawley (SD) rats were purchased from Sino-British SIPPR/BK Laboratory Animal Co. Ltd (Shanghai, China), housed in standard 80 polypropylene cages with sawdust bedding, and fed a pellet diet (Shanghai Shilin Science & Tech Co., Ltd., China) and water in glass bottles, ad libitum. The feed, water, and cages were all autoclaved, and the litter was changed every two days. The rats were housed in a room maintained at 20–26 °C at a humidity of 40–70% under a 12-h:12-h light/dark cycle. All animals were handled according to the Guidelines for the Care and Use of Laboratory Animals. All experimental procedures were approved by the Laboratory Animal Ethics Committee of the Shanghai Institute of Planned Parenthood Research. Fifty 3-month-old male rats were randomly divided into five groups (n = 10), and treated separately with vehicle and BPA (10, 30, 90, 270 µg/kg, daily gavage) for 4 weeks. All animals were weighed prior to being sacrificed. After the final administration of BPA, the blood from each animal was collected and the animals were sacrificed by decapitation. After the prostate glands were collected, the ventral and dorsolateral lobes were dissected and weighed. A section of the prostate gland was immediately fixed in 10% formalin, while the other was frozen in liquid nitrogen at − 80 °C until further use.

### Histological evaluation

The formalin-fixed tissues were embedded in paraffin, and then were cut into 3-μm-thick sections using a microtome. The sections were deparaffinized, rehydrated, and stained using hematoxylin and eosin (H&E). The slides were observed under a microscope (Nikon, Japan).

### ELISA detection

Blood was collected from the animals and centrifuged to collect serum. The prostate tissues were cut into pieces, ground with physiological saline (w:v = 1:10), and centrifuged to obtain the supernatant. The serum levels of testosterone (T) and estradiol (E_2_) and the levels of COX-2, L-PGDS, and PGFS in prostate tissues were measured using enzyme-linked immunosorbent assay kits (Testosterone/Estradiol ELISA Kits, Cayman, USA; PTGS2/ PGD2S/ PGFS ELISA Kits, MyBioSource, USA) and an enzyme-linked immunosorbent analyzer (Zenyth 200st, Austria).

### Immunohistochemistry

Immunohistochemical analysis of the paraffin sections of prostate tissues was performed to analyze the expressions of proliferating cell nuclear antigen (PCNA), androgen receptor (AR), estrogen receptor α (ERα), cyclooxygenase-2 (COX-2), L-type prostaglandin synthase (L-PGDS), and transcription factors nuclear factor κB (NF-κB). The sections were incubated with primary antibodies against PCNA (sc-56, Santa Cruz, USA), rabbit anti-AR (BA0004, Wuhan Boster, Wuhan, China), ER polyclonal (212441-1-AP, ProteinTech Group,Inc., USA), rabbit anti-rat COX-2 (sc-166475, Santa Cruz, USA), rabbit anti-rat L-PGDS (ab182141, Abcam, UK), and rabbit anti-rat NF-κB p65 (8242, Cell Signaling, USA), respectively, at a dilution of 1:100 overnight at 4 °C. The sections were then incubated with secondary antibody at 37 °C for 20 min. A negative control was establlished by omitting the use of primary antibodies. The experimental operations were perfomed according to those established in a previous study^8^. Six tissues sections were selected from each group, and ten microscopic fields for each section were randomly chosen was randomly observed. Semi-quantitative analysis of protein expression and the positive expression rate of PCNA were determined using Image-Pro Plus 6.0 to obtain the optical density of the slice.

### TUNEL assay

The rate of apoptosis in prostate tissues was detected using a TUNEL kits (rabbit polyclonal IgG, Abcam, UK). The paraffin sections were treated with proteinase K (20 µg/ml in 10 mM Tris HCl, pH8.0) for 30 min at room temperature. Endogenous peroxidases were blocked with 3% H_2_O_2_ for 5 min. Subsequently, the sections were incubated with biotinylated-TdT and enzyme TdT at 37 °C in the dark for 1 h. After the reaction was completed, the biotinylated nucleotides were detected using streptavidin peroxidase conjugate, stained with Harris’s hematoxylin for 5 min, and then stopped via the addition of water. The sections were dehydrated, washed, and mounted. The positive rate of apoptosis was determined using Image-Pro Plus 6.0.

### Western blot

Animal tissues were cut into pieces and homogenized with fivefold volumes of ice-cold RIPA lysate buffer (Weiao, China), followed by centrifugation to collect the supernatants. The cells were collected and RIPA lysate buffer (Weiao, China) was added to extract the protein. The total protein concentration was determined using a BCA kit per manufacturer’s protocol. The primary antibodies against COX-2 (sc-166475, Santa Cruz, USA; 1:800), L-PGDS (ab182141, Abcam, UK; 1:1000), NF-κB p65 (8242, Cell Signaling, USA; 1:1000) and GAPDH (WB0197, Weiao, China; 1:2000) were used to detect expressions of the COX-2, L-PGDS and NF-κB p65 proteins, respectively. The secondary antibodies included horseradish peroxidase-conjugated goat anti-rabbit or anti-mouse IgG (Jackson, USA; 1:2000). The membrane was stained with ECL reagent (Weiao, China), and the average optical density (IOD) of the bands in the membrane was detected using Image-Pro Plus 6.0. The relative expression of protein was equivalent to the ratio of the IOD of the protein band to that of the GAPDH band.

### Cell culture

Human prostate epithelial cells (HPEpiC) and human prostate fibroblasts (HPrF) were purchased from American Sciencell Company, and cultured in prostate epithelial cell medium and fibroblast medium (Sciencell) as described in manufacturer’s protocol.

### Cell viability

The cell viability of HPEpiC and HPrF was measured using a CCK-8 kit (DojinDo, Japan). HPEpiC at a density of 5,000 cells per well and HPrF at a density of 2,500 cells per well were seeded in 96-well plates and cultured for 24 h. To determine the effect of BPA on prostate cell proliferation, the cells in 96-well plates were incubated with BPA (0.01 nM–100 nM) diluted in cell medium containing 1‰ DMSO for 72 h. To study the effect of the PGS inhibitors, the cells were treated with 1‰ DMSO, BPA, BPA with COX-2 inhibitor, COX-2 inhibitor (NS398), BPA with L-PGDS inhibitor, and L-PGDS inhibitor (AT56) for 72 h. The CCK-8 reagents were diluted with cell medium (dilution, 1:9). Thereafter, the diluted mixture was added to the wells (100 µl per well). The cells were incubated at 37 °C for 1–4 h. The optical density (OD) was read using a microplate reader (Thermo Fisher Scientific, USA) at a wavelength of 450 nm; the cell viability (% of control) was calculated according to the manufacturer’s instructions.

### Apoptosis assay

HPEpiC at a density of 4 × 10^5^ cells per well and HPrF at a density of 2 × 10^5^ cells per well were seeded in 6-well plates and cultured for 24 h. Thereafter, the cells were treated with 1‰ DMSO, BPA, BPA with COX-2 inhibitor, COX-2 inhibitor (NS398), BPA with L-PGDS inhibitor, and L-PGDS inhibitor (AT56) for 72 h. The rate of apoptosis was detected using the FITC Annexin V apoptosis detection kit (BD, USA), and then measured via flow cytometry (Beckman, USA).

### Statistical analysis

Statistical comparisons were performed using one-factor analysis of variance (ANOVA). If statistically significant, the differences in the planned comparisons between the control and treatment groups were derived using Dunnett’s least-squares means test. Data were analyzed using SPSS version 23.0 (SPSS, IL, USA) and are presented as mean ± standard deviation.

## Results

### BPA causes imbalance in cell proliferation and apoptosis and induces prostatic hyperplasia in adult rats

**Figure 1:**
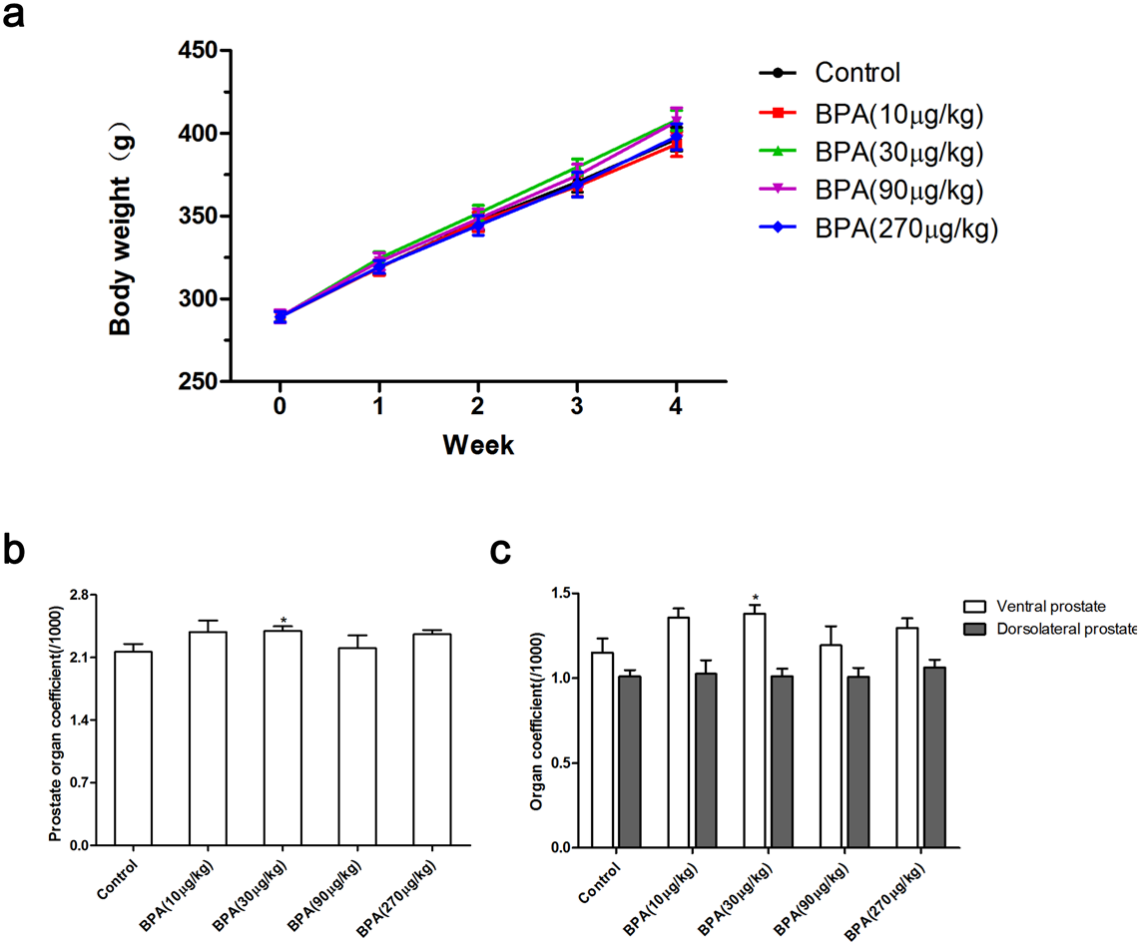
Effect of BPA on body weight and prostate organ coefficient in adult rats. (**a**) The change in weight of rats during the 4-week period. Effect of BPA on the organ coefficient of prostate (**b**) and all lobes of the prostate (**c**). BPA: Bisphenol A. Values are presented as mean ± SD. Organ coefficient of DLP = The weight of DLP/Terminal body weight × 1,000. (n = 10, *P < 0.05 compared to control).

During the 4-week administration period, BPA within the dose range had no significant effect on the weight gain of rats. The organ coefficients of the whole prostate and the ventral prostate were significantly increased in the BPA (30 µg/kg)-treated group compared to the control (P < 0.05) (Fig. 1).

**Figure 2:**
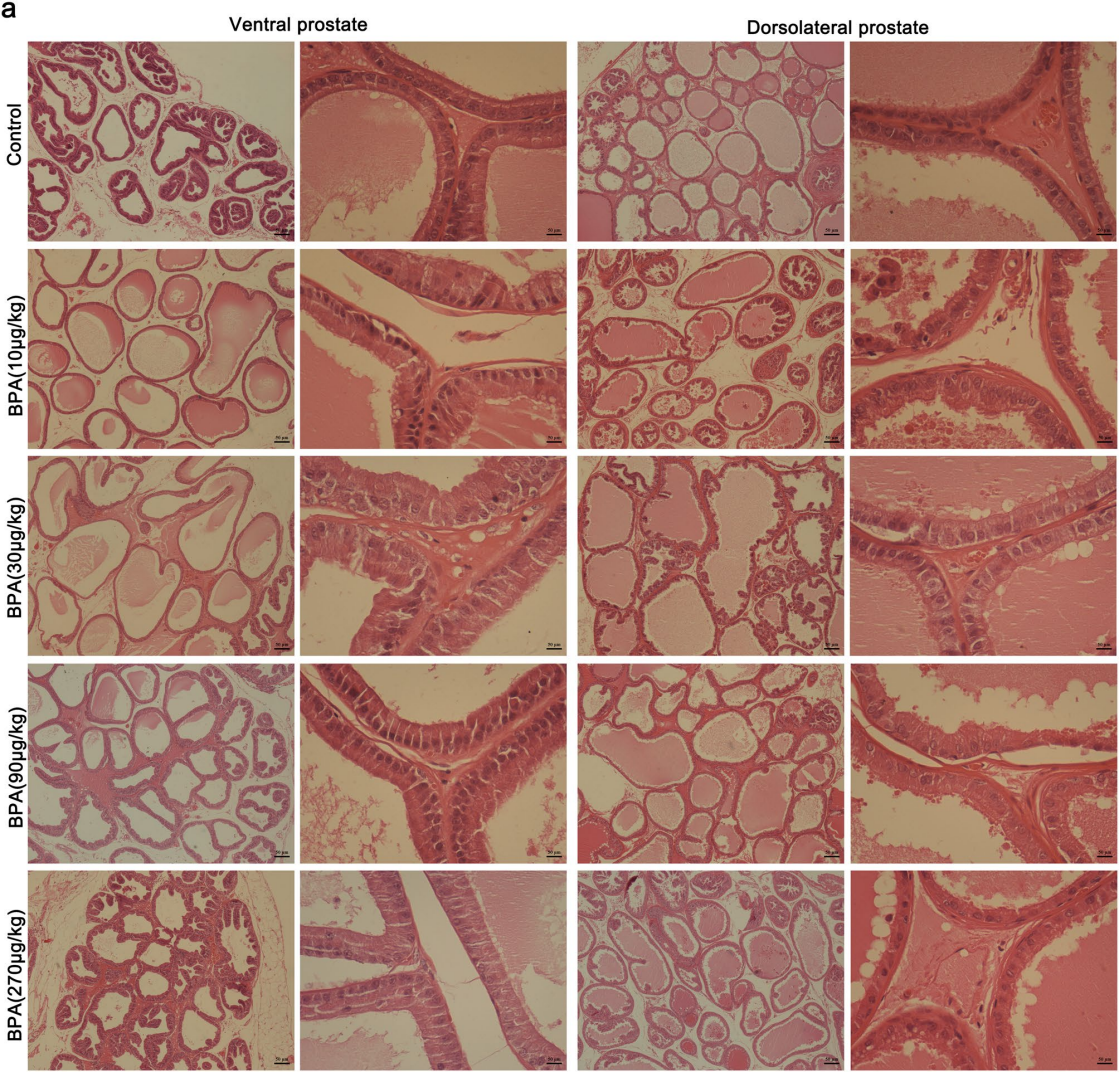
Histological staining of the prostate in adult rats after 4-weeks of BPA treatment (scale bar = 50 µm; magnification × 40, × 400).

Based on the results of H&E staining, the gland cavity of ventral and dorsolateral prostates in BPA-treated group sufficiently expanded compared to the control group. The glandular secretions were increased in the BPA-treated groups. The epithelial height of the prostate samples in the BPA-treated groups was higher than that in the control group (Fig. 2).

**Figure 3:**
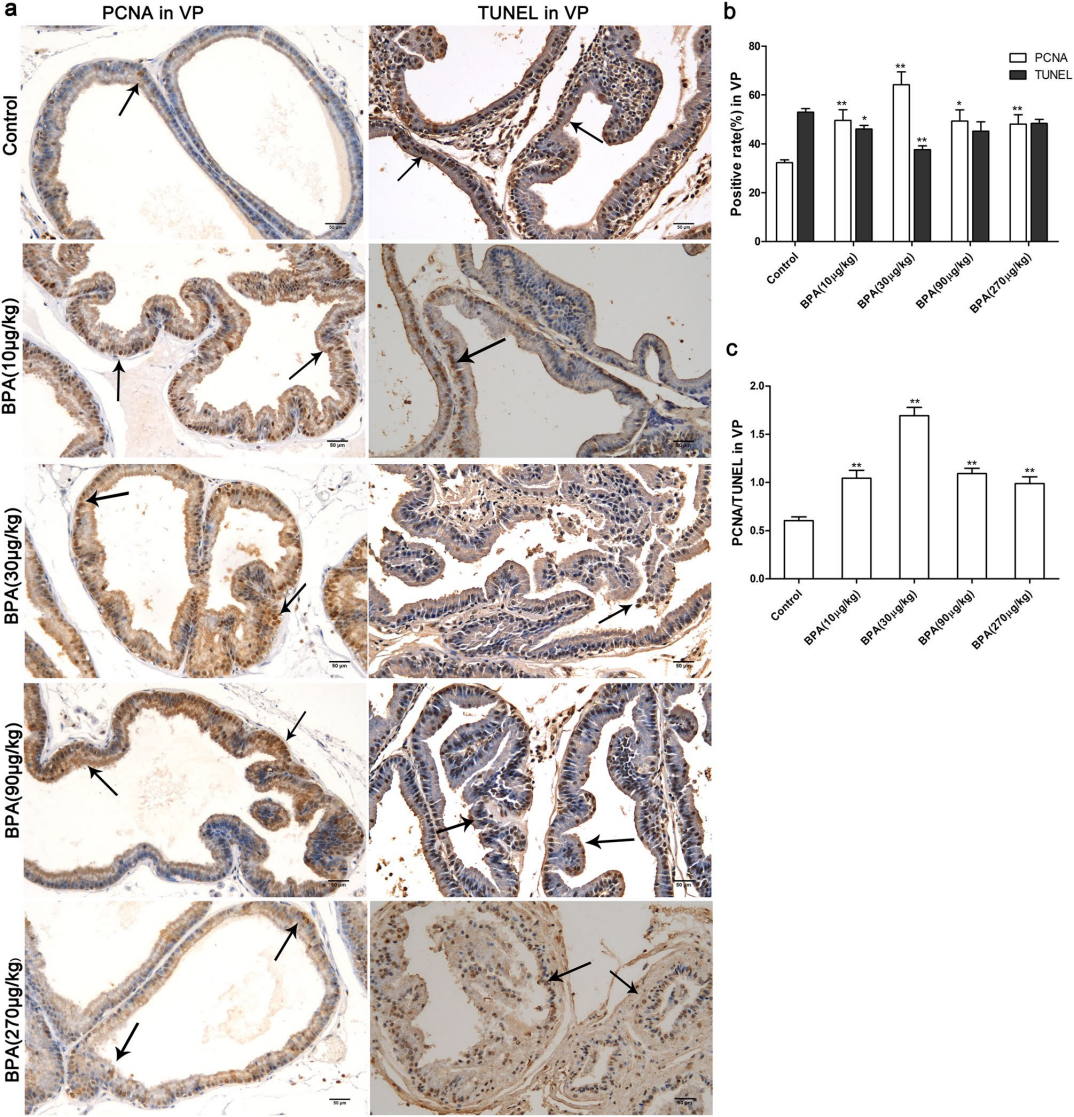
Effect of BPA on cell proliferation and death in the ventral prostate of adult rats. (**a**) Immunohistochemical images of PCNA and TUNEL in the ventral prostate; (**b**) Effect of BPA on PCNA-positive ratio and TUNEL-positive ratio in the ventral prostate; (**c**) Effect of BPA on the ratio of PCNA/TUNEL in the ventral prostate. VP: Ventral prostate. Scale bar = 50 µm, magnification × 200. Values are presented as mean ± SD (n = 4, *P < 0.05, *P < 0.01, compared to the control).

**Figure 4:**
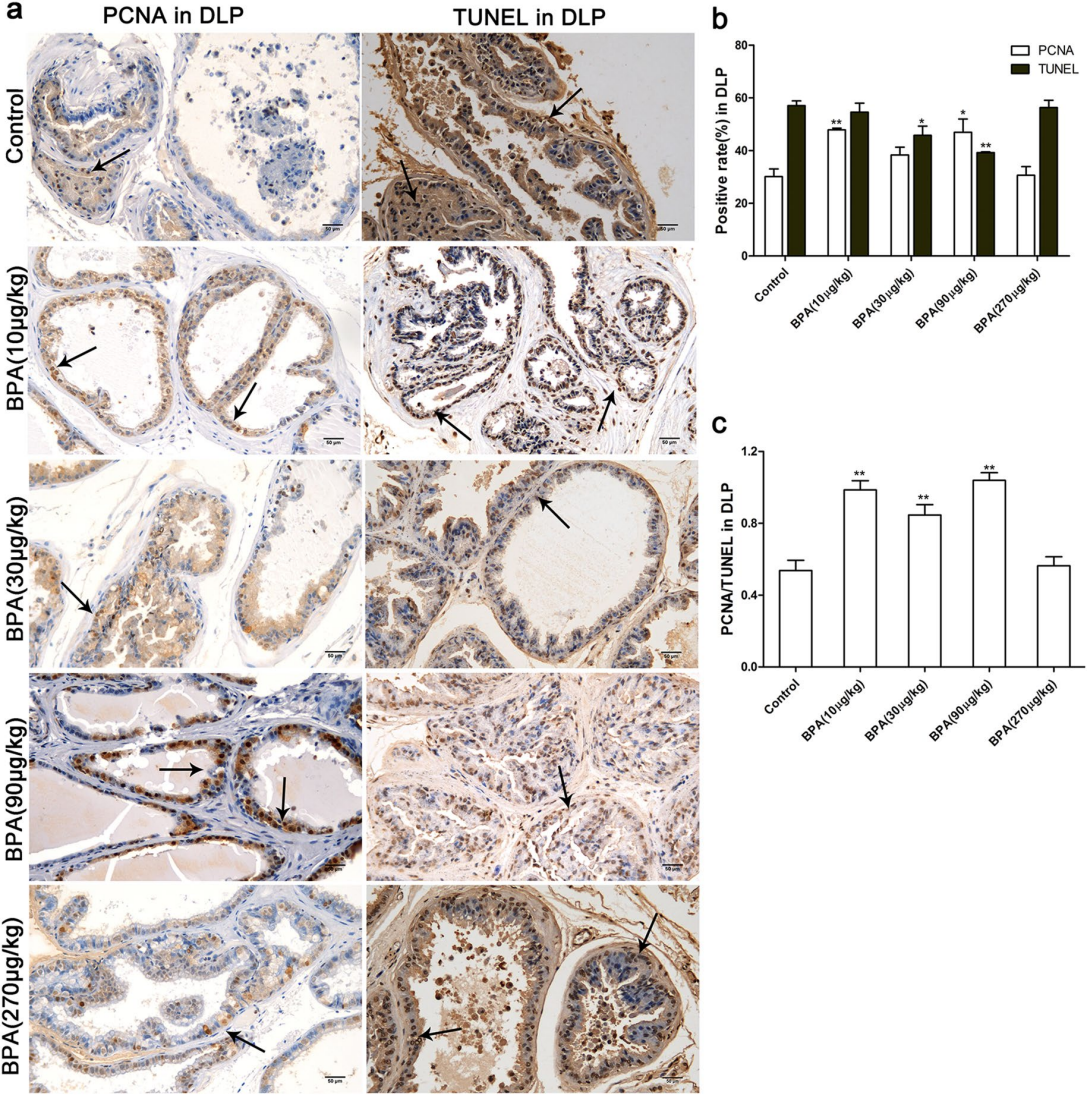
Effect of BPA on cell proliferation and death in the dorsolateral prostate of adult rats. (**a**) Immunohistochemical images of PCNA and TUNEL in the dorsolateral prostate; (**b**) Effect of BPA on PCNA-positive ratio and TUNEL-positive ratio in the dorsolateral prostate; (**c**) Effect of BPA on the ratio of PCNA/TUNEL in the dorsolateral prostate. DLP: dorsolateral prostate. Scale bar = 50 µm, magnification × 200. Values are presented as mean ± SD (n = 4, *P < 0.05, *P < 0.01, compared to the control).

PCNA is a cell-proliferation antigen that is mainly expressed in the nucleus. Herein, a TUNEL assay was performed to detect apoptosis in the tissues. Cell proliferation and apoptosis in tissues were measured using the ratio of PCNA to TUNEL^22^. Compared to the control, the positive rate of PCNA in the ventral prostates of the BPA group was significantly increased (P < 0.01), and reached a maximum in the BPA (30 µg/kg) group (P < 0.01). The apoptotic rate of ventral prostates was significantly decreased in the low-dose BPA groups (10 and 30 µg/kg) (P < 0.01). Thus, the PCNA/TUNEL ratio in the ventral prostate increased after BPA administration (P < 0.01), and reached a peak in the BPA (30 µg/kg)-treated group (Fig. 3). The changing trends of the PCNA-positive and TUNEL-positive rates after BPA treatment in the dorsolateral prostate were similar to those in the ventral prostate. Compared to the control, the PCNA/TUNEL ratio of dorsolateral prostate was increased in the BPA (30–90 µg/kg)-treated groups (P < 0.01) and decreased in the high-dose BPA-treated group (270 µg/kg) to the level of the control (Fig. 4).

### BPA treatment alters the E_2_ and T levels in the serum and the contents of prostaglandin synthases in the prostate

**Figure 5:**
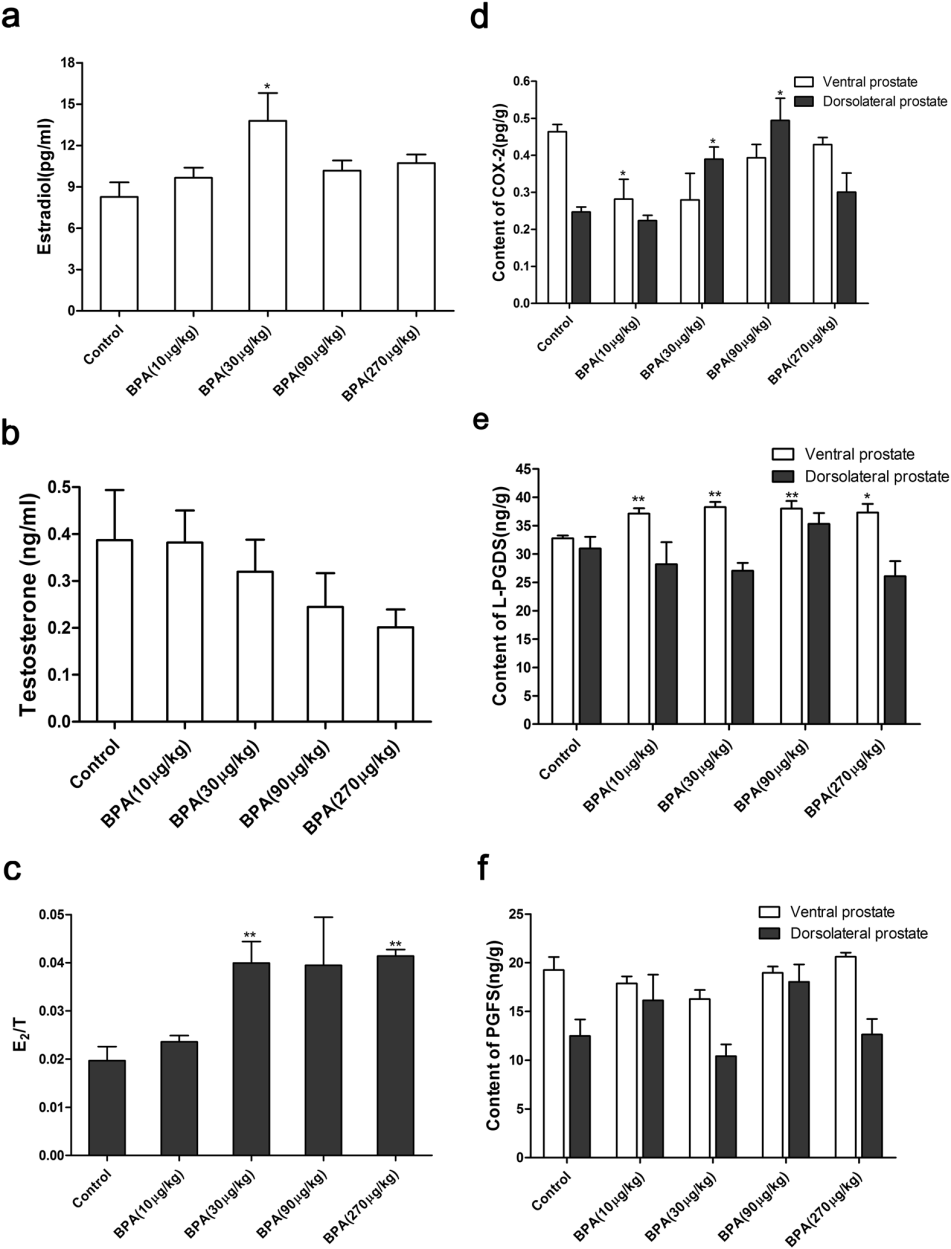
Effect of BPA on the serum levels of E_2_ and T and the content of COX-2, L-PGDS, and PGFS in the prostate of adult rats. E_2_ (**a**) and T (**b**) levels in serum, and E_2_/T ratio (**c**); (**d**–**f**) COX-2, L-PGDS, and PGFS content in the prostate. Values are presented as mean ± SD (n = 6, *P < 0.05, *P < 0.01, compared to the control).

As shown in Fig. 5a–c, the serum levels of E_2_ in adult rats were significantly increased in the BPA (30 µg/kg)-treated group due to BPA administration for 4 weeks (P < 0.05) while the testosterone levels in serum were slightly decreased in the BPA-treated group. The ratio of E_2_ to T was significantly increased in the BPA (30 μg/kg and 270 μg/kg)-treated groups (P < 0.01). As shown in Fig. 5d–f, the COX-2 level in the ventral prostate in the BPA-treated group was lower than that in the control group while that of the dorsolateral prostate was increased in the BPA (30 μg/kg and 90 μg/kg)-treated groups (P < 0.05). The L-PGDS level of the ventral prostate in the BPA-treated groups was significantly higher than that in the control group (P < 0.01) and reached a maximum in the BPA (30 μg/kg)-treated group. The L-PGDS content of the dorsolateral prostate showed no significant change. No significant alteration of the PGFS content in the ventral and dorsolateral prostates was observed upon treatment with BPA.

### BPA induces a higher expression of AR and ERα.

**Figure 6:**
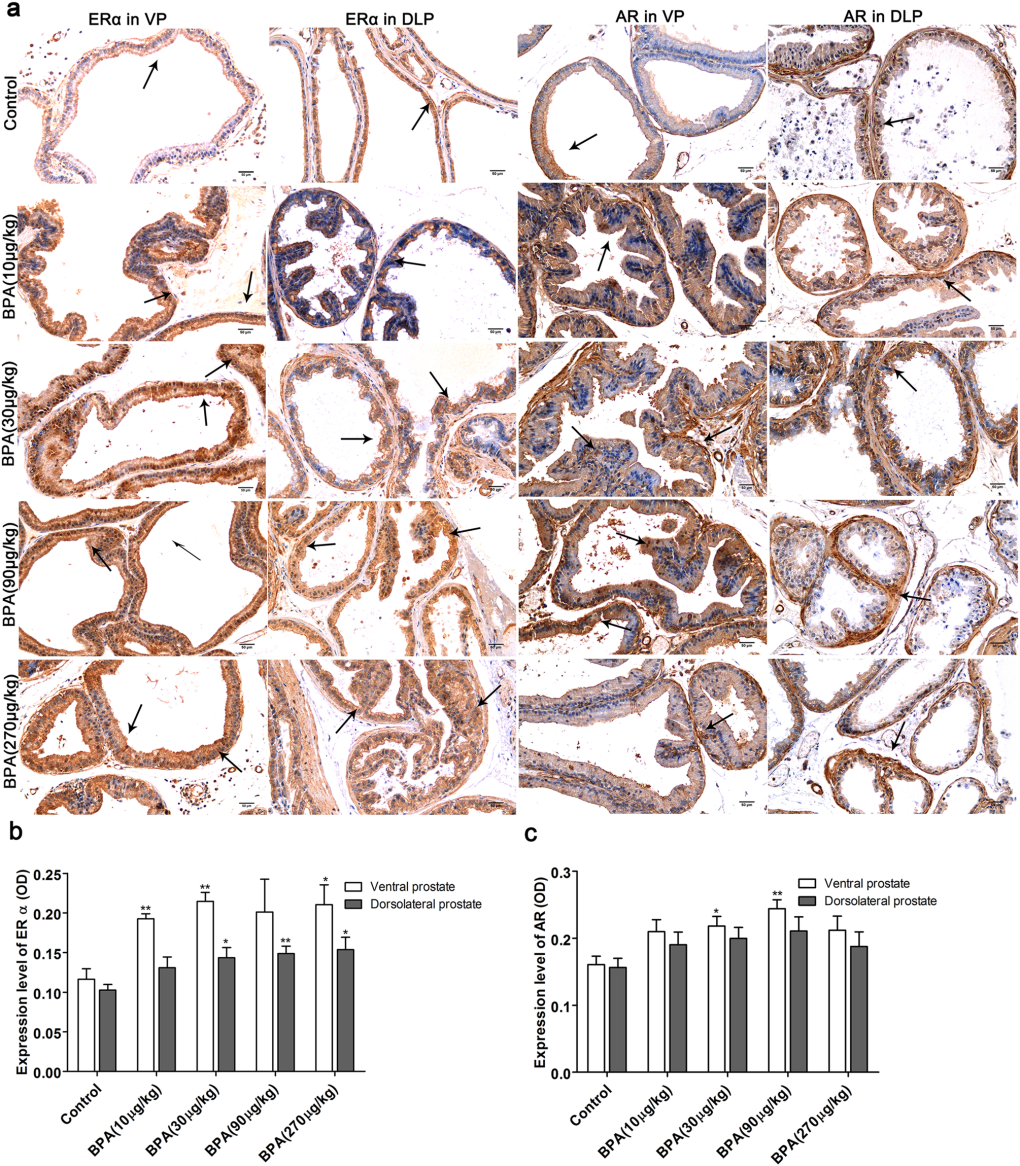
Immunohistochemical analysis of ERα and AR in the prostate of adult rats. (**a**) Immunohistochemical images of ERα and AR in the ventral and dorsolateral prostates; Effect of BPA on ERα and AR expression in the ventral prostate (**b**) and dorsolateral prostate (**c**). VP: ventral prostate; DLP: dorsal prostate. Scale bar = 50 µm, magnification × 200. Values are presented as mean ± SD (n = 4, *P < 0.05, *P < 0.01, compared to the control).

The results of immunohistochemical analysis (Fig. 6) revealed that ERα expression in the ventral prostate was increased in groups treated with BPA (10 and 30 µg/kg, P < 0.01 and 270 µg/kg, P < 0.05) compared to the control. The ERα expression level in the dorsolateral prostate increased as the BPA dose (30–270 µg/kg) increased (P < 0.05). Compared to the control, AR expression in the ventral prostate was increased in groups treated with BPA (30 µg/kg, P < 0.05 and 90 µg/kg, P < 0.01) while that in the dorsolateral prostate of the BPA-treated groups showed no significant difference.

### Low-dose BPA increases the expression of L-PGDS, COX-2 and NF-κB

**Figure 7:**
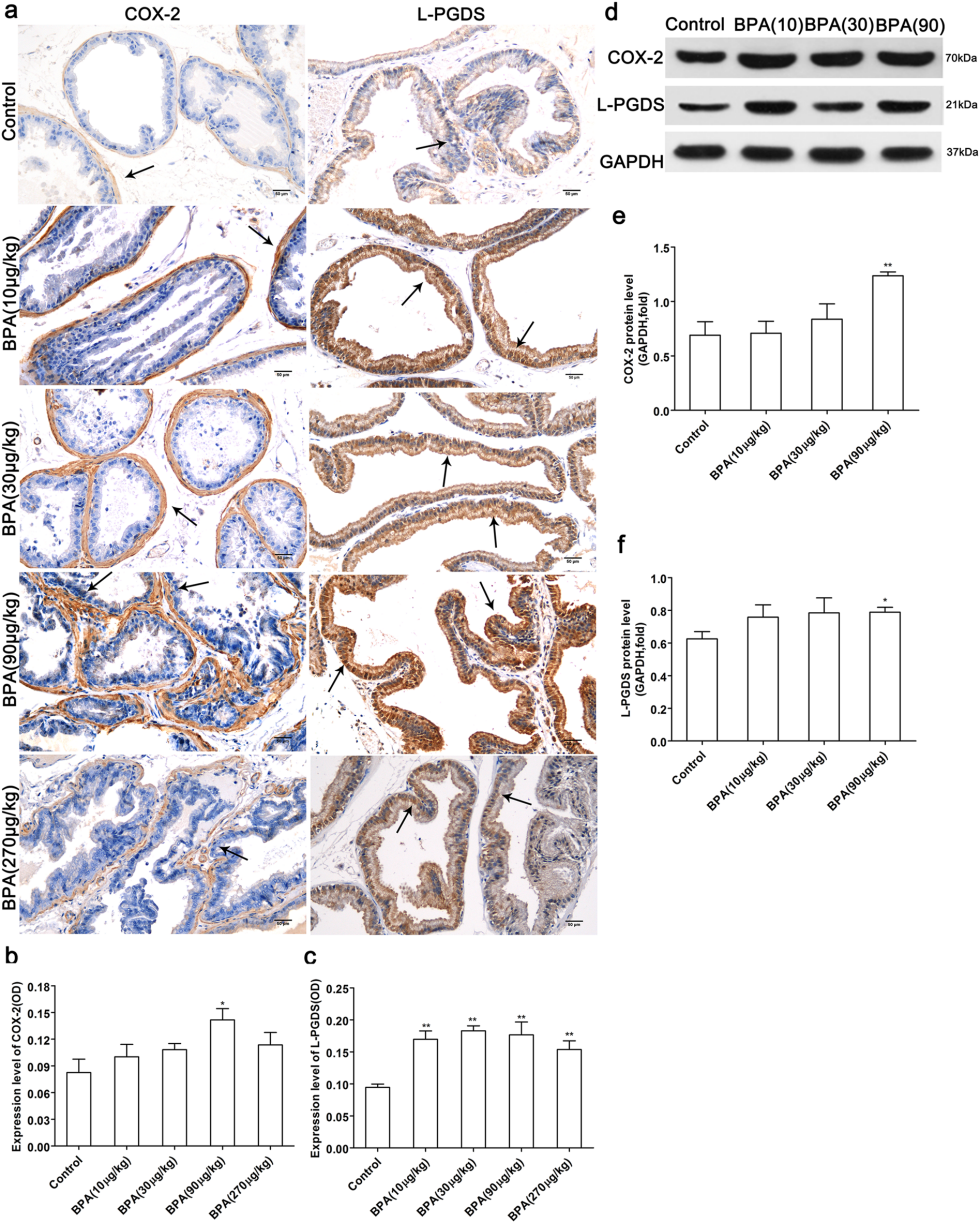
Effect of BPA on the expression of COX-2 and L-PGDS in the prostate. (**a**) Immunohistochemical images of COX-2 in the dorsolateral prostate and L-PGDS in the ventral prostate; Effect of BPA on COX-2 (**b**) and L-PGDS (**c**) expression in the prostate. (Scale bar = 50 µm, magnification × 200, n = 4). The protein bands of COX-2 and L-PGDS (**d**); The expression levels of the COX-2 protein in the dorsolateral prostate (**e**) and L-PGDS protein in the ventral prostate (**f**). Blots (**d**) were cropped from the gel presented in Supplementary Fig. 1. Bands were quantified using densitometry, with the results normalized to GAPDH expression in each sample (n = 3). Values are presented as mean ± SD (*P < 0.05, **P < 0.01, compared to the control).

As shown in Fig. 7, the expression level of COX-2 in the dorsolateral prostate of the BPA-treated group was higher than that in the control group and significantly increased in the BPA (90 µg/kg)-treated group (P < 0.05). In addition, the expression level of L-PGDS was increased in the BPA-treated groups (P < 0.01). The results of western blotting indicated that the relative expression levels of the L-PGDS protein in the ventral prostate and COX-2 protein in the dorsolateral prostate were increased in the BPA (90 µg/kg)-treated groups relative to the control group (P < 0.05, P < 0.01).

**Figure 8:**
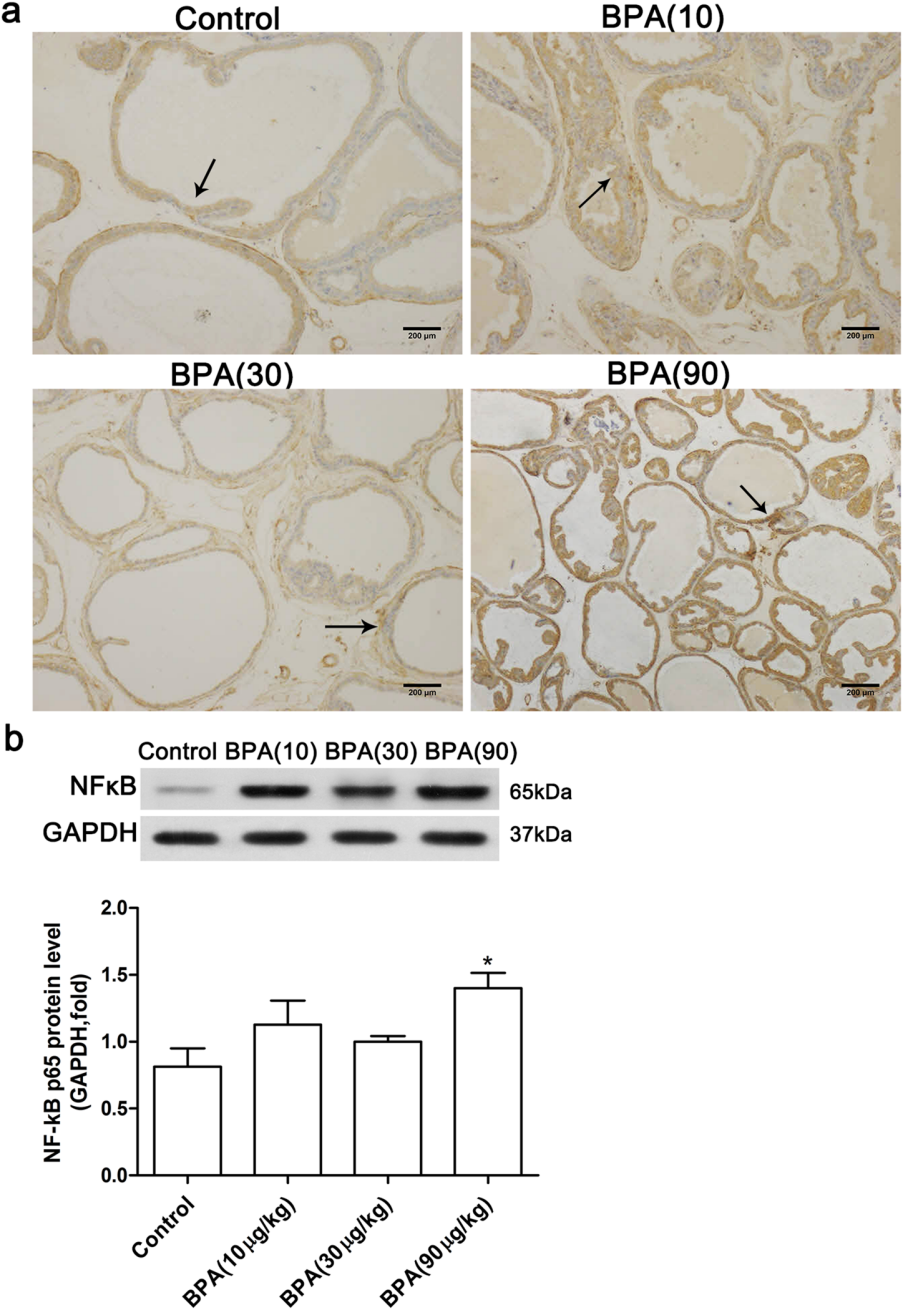
Effect of BPA on NF-κB p65 expression in the dorsolateral prostate. (**a**) Immunohistochemical staining of NF-κB in the prostate (Scale bar = 200 µm, magnification × 100, n = 4); (**b**) the protein band and the expression level of the NF-κB p65 protein. Bands were quantified using densitometry, with the results normalized to GAPDH expression in each sample (n = 3). Values are presented as mean ± SD (*P < 0.05, compared to the control).

Low-dose BPA treatment increased the expression of NF-κB p65 in the dorsolateral prostate (Fig. 8). Immunohistochemical staining (Fig. 8a) showed that BPA treatment induced an increase of the NF-κB p65 expression in the dorsolateral prostate. The results of western blotting indicated that the NF-κB p65 protein level was significantly increased in the BPA (90 µg/kg)—treated group (P < 0.05) compared to the control.

### Low-dose BPA promotes prostate cell proliferation and up-regulates the expression of COX-2 and L-PGDS

**Figure 9:**
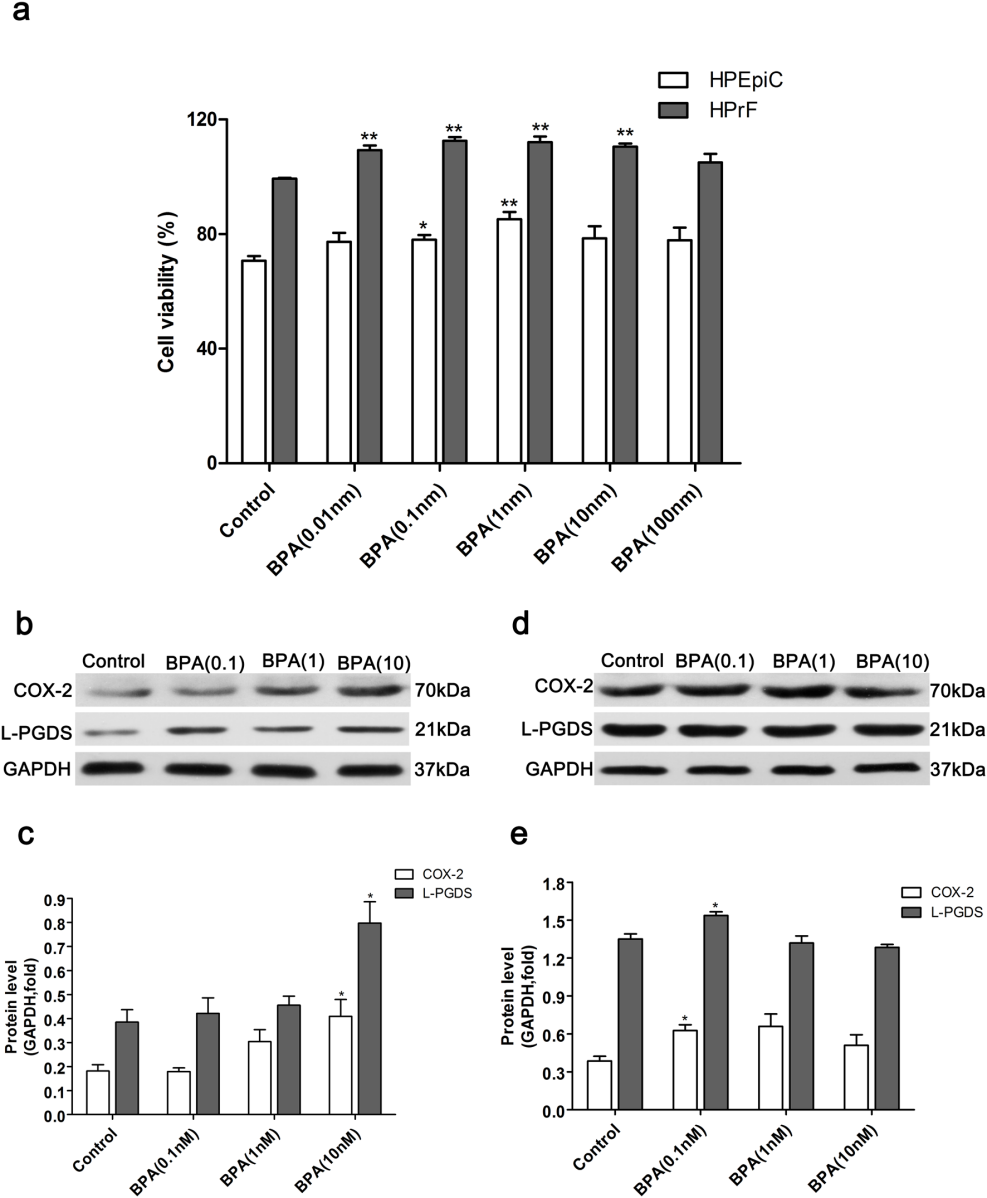
Effect of BPA on the viability of human prostate epithelial cells and human prostate fibroblasts and the expression of COX-2 and L-PGDS proteins in prostate cells. (**a**) Effect of BPA on the viability of HPEpiC and HPrF. The protein bands of COX-2 and L-PGDS in HPEpiC (**b**) and HPrF (**d**). The expression levels of the COX-2 and L-PGDS proteins in HPEpiC (**c**) and HPrF (**e**). Blots (**b**, **d**) were cropped from the gel presented in Supplementary Figure S2. Bands were quantified using densitometry, with results normalized to GAPDH expression in each sample. HPEpiC: human prostate epithelial cells; HPrF:human prostate fibroblasts. Values are presented as mean ± SD (n = 3, *P < 0.05, **P < 0.01, compared to the control).

CCK-8 detection results showed that the viability of human prostate fibroblasts in the BPA-treated group was increased (P < 0.01) relative to the control and reached a maximum in the BPA-treated group (0.1 nM). The viability of human prostate epithelial cells in the BPA-treated groups was also increased (P < 0.01). A similar trend was observed for prostate fibroblasts and reached a maximum with BPA (1 nM) (Fig. 9a). Thus, 0.1 nM and 1 nM were considered as optimal doses of BPA to promote the proliferation of prostate fibroblasts and prostate epithelial cells.

Results of western blotting revealed that 0.1–10 nM BPA upregulated the expression of COX-2 and L-PGDS in prostate epithelial cells in a dose-dependent manner. Significant proliferation of prostate fibroblasts and prostate epithelial cells was observed in BPA (10 nM)-treated group (P < 0.05). The expression of COX-2 and L-PGDS in prostate fibroblasts was increased in BPA (0.01 nM)-treated group (P < 0.05, Fig. 9).

### Inhibition of COX-2 and L-PGDS inhibits BPA-induced prostate cell proliferation and promotes apoptosis

**Figure 10:**
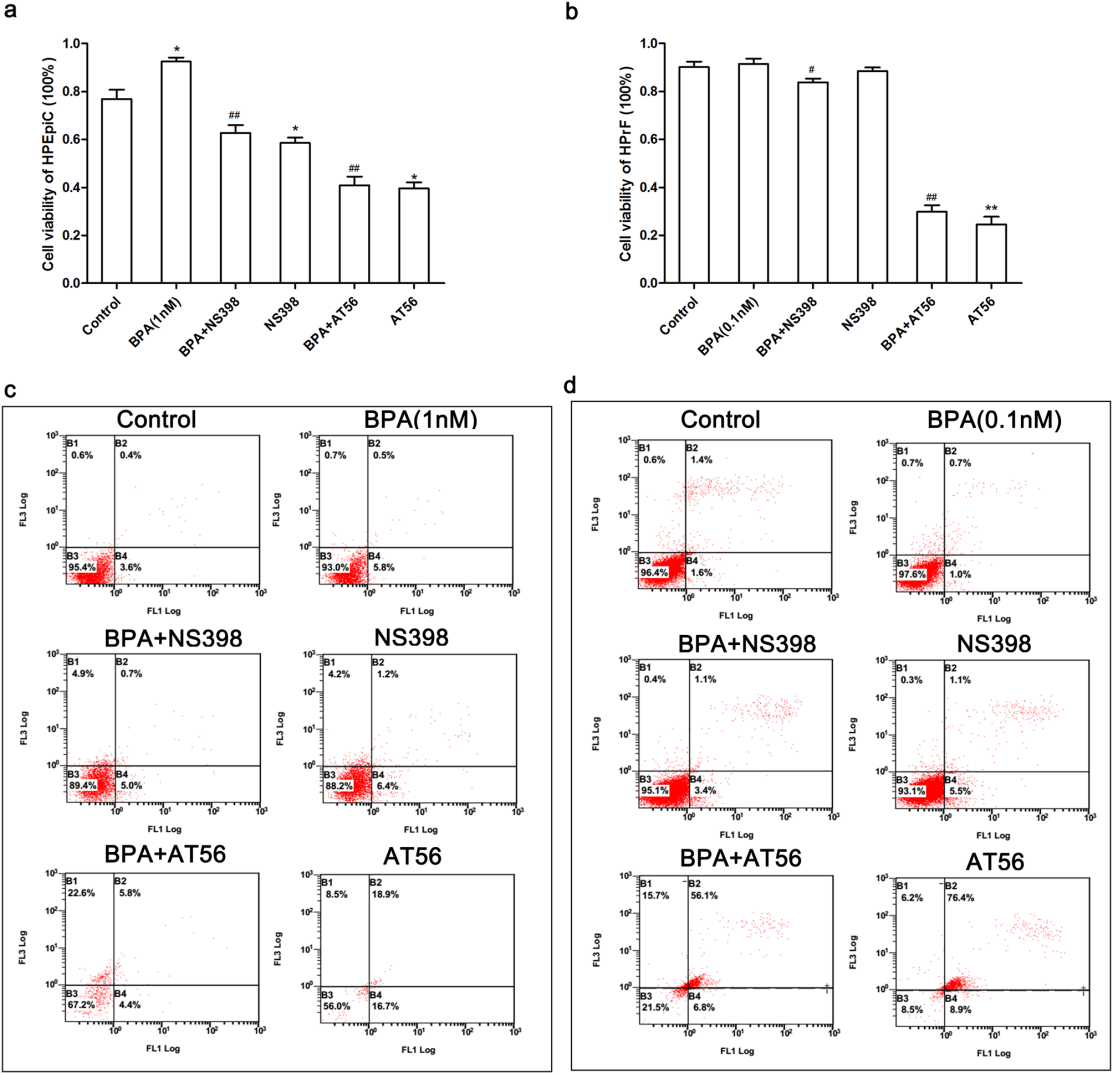
COX-2 and L-PGDS inhibitors suppressed the proliferation of prostate cells induced by BPA and promoted apoptosis. Effect of the COX-2 and L-PGDS inhibitors on the viability of HPEpiC (**a**) and HPrF (**b**); Effect of the COX-2 and L-PGDS inhibitors on HPEpiC (**c**) and HPrF (**d**). (HPEpiC: human prostate epithelial cells; HPrF: human prostate fibroblasts. FL1: FITC channel, FL3: PI channel; B2: FITC+/PI+, late stage apoptotic cells; B3: FITC-/PI−, normal cells; B4: FITC+/PI−, viable apoptotic cells; NS398: COX-2 inhibitor, AT56: L-PGD inhibitor).

To determine the effects of the prostate synthase inhibitors on BPA-induced prostate cell proliferation, a CCK-8 assay was used to detect the viability of prostate cells treated with BPA and prostate synthase inhibitors. The apoptotic rate was determined using FITC AV-PI double staining and flow cytometry. As seen in Fig. 10, compared to the BPA-treated group, the COX-2 specific inhibitor (NS398) and L-PGDS specific inhibitor (AT56) significantly inhibited the prostate epithelial cell proliferation induced by BPA (1 nM) (P < 0.01, Fig. 10a). Compared to the BPA (0.1 nM)-treated group, the cell viability of prostate fibroblasts was significantly decreased by NS398 (P < 0.05) and AT56 (P < 0.01) (Fig. 10b). Both NS398 and AT56 inhibited the growth of prostate epithelial cells; however, the inhibitory effects of AT56 were more potent. The apoptotic rate of prostate epithelial cells treated with BPA was lower than that of the control. The administration of BPA combined with NS398 or AT56 increased the apoptotic rate of prostate cells compared to the BPA-treated group.

## Discussion

In vivo studies demonstrated that adult SD rats treated with 10–270 μg/kg BPA for 4 weeks could suffer from prostatic hyperplasia, and in vitro studies indicated that low-dose BPA (0.1 nM and 1 nM) significantly promoted the proliferation of prostate fibroblasts and epithelial cells in humans. The PGS COX-2 and L-PGDS showed significant upregulation in both in vivo prostatic hyperplasia and in vitro prostate cell proliferation induced by low-dose BPA. In vitro data further supported that COX-2 and L-PGDS play a critical role in prostate cell proliferation.

Environmental exposure to BPA exerted low-dose effects of promoting proliferation on the prostate. Results from animal experiments indicated that a 1–2 μg/(kg d) exposure level of BPA in the environment^1^, and a daily safe dose of 50 μg/(kg d)^23^ could increase the number and volume of the dorsolateral prostate in the mice offsprings^3^ as well as the weight of the ventral prostate in rats^24^. This study further confirmed that a daily administration of 10–270 μg/kg BPA in adult rats could induce prostatic hyperplasia, which is characterized by the increased organ coefficient of prostate, PCNA expression and the ratio of proliferation/apoptosis. To study the low-dose effects of BPA on the prostate in vitro, two prostate cell lines representing the prostate fibroblasts and epithelial cells were used. The BPA concentration in vitro is generally lower than 100 nM (10^−7^ M)^25^. Further, 1–10 nM BPA disturbs the early morphogenesis of human prostate stem cells in a dose-dependent manner^26^ and promotes the migration of prostate cancer cells^27^. This study found that BPA (0.01–100 nM) promoted the proliferation of normal human prostate epithelial cells and fibroblasts, and decreased apoptosis.

An imbalance in cell proliferation and apoptosis is a critical pathogenesis of prostate hyperplasia. In vivo PCNA and TUNEL assays showed that with an increase in the BPA dose, the ratio of proliferation to apoptosis in the ventral and dorsolateral prostates initially increased and then decreased, indicating that low-dose BPA induced prostate hyperplasia by promoting the proliferation of prostate cells. However, the increased apoptosis induced by high-dose BPA treatment might attenuate the trend of hyperplasia^28^. Prostate is a hormone-sensitive organ, and the interaction between estrogen and androgen is essential to maintain its normal development^29,30^. As a non-steroidal estrogen, BPA can disturb the expression of hormone receptors in some target tissues and affecte the endogenous hormone activity. By binding to the estrogen receptors ERα and ERβ, BPA regulates estrogen signaling pathways and the expression of target genes. ERα mediates BPA-induced prostate cell proliferation^25^, while ERβ mediates apoptosis induced by high-dose BPA^7^, stimulating the ERK-dependent signaling pathway to induce the expression of p53 through a pathway involving the ERβ/EGFR complex^31^. High-dose BPA has strong anti-androgenic activity^32^, while low-dose BPA can promote AR expression in prostate cells^33^. Our study revealed that low-dose BPA could significantly promote the expression of AR in the ventral prostate. When the ratio of estrogen to androgen is increased, the upregulated AR expression might increase the sensitivity of the prostate to androgens, thereby promoting prostate hyperplasia.

COX-2 and L-PGDS play critical roles in maintaining normal physiological function. Abnormal expression is associated with pathological changes in normal systems including the male reproductive system. In this study, we found that low-dose BPA upregulated COX-2 and L-PGDS expression and induced prostatic hyperplasia; however, their inhibition attenuated the effects of BPA. The regulation of COX-2 by BPA might be related to the interaction of COX-2 and estrogen. In various estrogen-dependent tumors including endometrial cancer and ovarian cancer, estrogen promotes the proliferation and invasion of cancer by upregulating COX-2 expression^34,35^. COX-2 is involved in the positive feedback regulation between estrogen and inflammatory factors, where COX-2/mPGES-1 upregulates the expression of aromatase and increases the levels of estrogen, which in turn upregulates COX-2 expression via combination with the estrogen receptor^36^. Low-dose BPA also upregulates aromatase, which catalyzes the production of estradiol from testosterone to increase the estradiol levels in adult rats^37^. In the male reproductive system, COX-2 inhibits the expression of the steroid-derived acute regulatory protein (StAR) gene in Leydig cells and induces an age-related decrease in testosterone levels^38^. Thus, BPA might directly induce COX-2 expression to increase E_2_ secretion and decrease T levels, while the increased estrogen in turn upregulates COX-2 expression in the prostate. Previous studies reveal that BPA induces the invasion of prostate cancer and hyperplasia of the dorsolateral prostate via the epithelial-mesenchymal transition (EMT) pathway^5,39^. The EMT pathway activated by COX-2^40^ is involved in the inflammatory response^41^. In addition, the upregulation of the NF-κB/COX-2/PGE pathway^11^ mediates the proliferation of cells. NF-κB activation was triggered by extracellular stimulation to upregulate many intracellular downstream signaling pathways including COX-2/PGE. Previous studies report that DHT treatment, in vivo or ex vivo, induces nuclear NF-κB activation and increases the levels of proinflammatory products of NF-κB activation including COX-2^42^. The dysregulation of NF-κB can induce autoimmune diseases, chronic inflammation, and cancers^43^. The upregulated NF-κB and its induced signaling pathways are involved in the development of prostate cancer and prostatitis^44,45^. GHRH stimulates the NFκB p65 pathway, which is involved in inflammation and growth in both BPH-1 and PrEp cells. Conversely, the GHRH antagonist significantly reduces these effects and decreases the inflammatory marker, COX-2^46^. BPA increases the expression of the proinflammatory mediator, PGE2, and its upstream factor, COX-2, with significant induction of phosphorylation and nuclear translocation of NF-κB p65^47^. The activation of NF-κB by BPA promotes an invasion process in breast cancer cells^48^. Additionally, low-dose BPA exerts its inhibition on apoptosis via increased anti-apoptotic protein Bcl-2 level^49^ and upregulates the phosphorylation of NF-κB p65^50^. Decreased COX-2 levels or COX-2 inhibitors potentiated apoptosis, which is related to the suppression of gene products associated with cell apoptosis (Bcl-2 and Bax)^51^. Thus, the upregulated NF-κB p65/COX-2 in the prostate might mediate BPA-inhibited apoptosis. The role of the NF-κB/COX-2 pathway in the effect of low-dose BPA on cell proliferation and apoptosis in the prostate deserves further research.

L-PGDS, which is mainly distributed in the Leydig cells of the testis and epithelial cells of the prostate and epididymis^52^, is associated with the development of male reproductive organs. The function of L-PGDS in reproductive organs is affected by androgen levels. Testosterone and testicular secretion factors in the epididymis regulate the synthesis and secretion of L-PGDS^53^. High-level androgens in male androgen alopecia (AGA) increase the expression of L-PGDS and PGD_2_ produced by L-PGDS catalysis^54^. Low-dose BPA promotes AR expression in prostate cells^32^. In this study, BPA was found to significantly increase AR and L-PGDS expression in the ventral prostate. Thus, under the conditions of increased estrogen/androgen ratio, low-dose BPA might increase L-PGDS expression by upregulating AR-induced androgen sensitivity of the ventral prostate. Studies have shown that L-PGDS is highly expressed in AGA, which is associated with a high incidence of BPH^55^. However, the role of L-PGDS in BPH remains unclear. PGDS is a bifunctional protein that catalyzes the synthesis of PGD and transports multiple lipophilic substances^52^. During PGFS catalysis, the PGD2 produced by L-PGDS catalysis is converted to PGF2, which is bound to the FP receptor to promote cell proliferation by activating the growth factor-dependent MAP kinase pathway^21^. In vitro studies have indicated that L-PGDS mediates cell proliferation via its lipid-carrying function in different cells. In human epidermal melanocytes, normal L-PGDS levels restrict the growth of epidermal melanocytes by transporting all-trans retinoic acid (RA), while its overexpression dysregulates the proliferation of melanoma cells^56^. Additionally, the protein L-PGDS accelerates the migration of glial cells and promotes reactive gliosis by interacting with protein kinase C substrate (MARCKS)^57^. Therefore, we hypothesized that L-PGDS is an apolipoprotein in the prostate that might contribute to the accumulation of the liposoluble substance, BPA, in and around prostate cells. L-PGDS may also assist BPA in promoting proliferation..

## Conclusion

This study is the first to reveal the functions of COX-2 and L-PGDS in prostatic tissues. PGS can be upregulated by low-dose BPA treatment to mediate BPA-induced prostatic hyperplasia. COX-2 and ERα, which are regulated by estrogen, might be involved in BPA-induced hyperplasia of the dorsolateral prostate via the activation of signaling pathways, including COX-2/NF-κB pathway, that promote prostate cell proliferation. L-PGDS, the target gene of androgen/AR, mediates BPA-induced hyperplasia of the ventral prostate; however, the underlying mechanism of the modulation of L-PGDS/PGD in cell proliferation warrants further research.

## Supplementary information

Supplementary information.
