# Supplementary material for: The prostaglandin synthases, COX-2 and L-PGDS, mediate prostate hyperplasia induced by low-dose bisphenol A
Source: Sci Rep. 2020 Aug 4;10:13108. doi: 10.1038/s41598-020-69809-y (PMC7403327; doi:10.1038/s41598-020-69809-y)
Supplement: Supplementary file 1 — Supplementary information. [file 41598_2020_69809_MOESM1_ESM.pdf]

Supplementary Information

The prostaglandin synthases, COX-2 and L-PGDS, mediate prostate hyperplasia induced by low-dose bisphenol A

Shuangshuang Wu[1,2,3,4], *Dongyan Huang*[2,3,4], *Xin Su*[2,3,4], *Han Yan*[2,3,4], *Aicui Ma*[2,3,4], *Lei Li*[2,3,4], *Jianhui Wu*[2,3,4*], *and Zuyue Sun* [2,3,4]

1. Pharmacy School of Fudan University, Shanghai 201203, China; 2. National Evaluation Centre for Toxicology of Fertility Regulating Drugs, Shanghai Institute of Planned Parenthood Research, Shanghai 200032, China; 3. Key Laboratory of Reproduction Regulation of NPFPC,Shanghai 200032, China; 4. Reproductive and Developmental Research Institute of Fudan University, Shanghai 200032, China

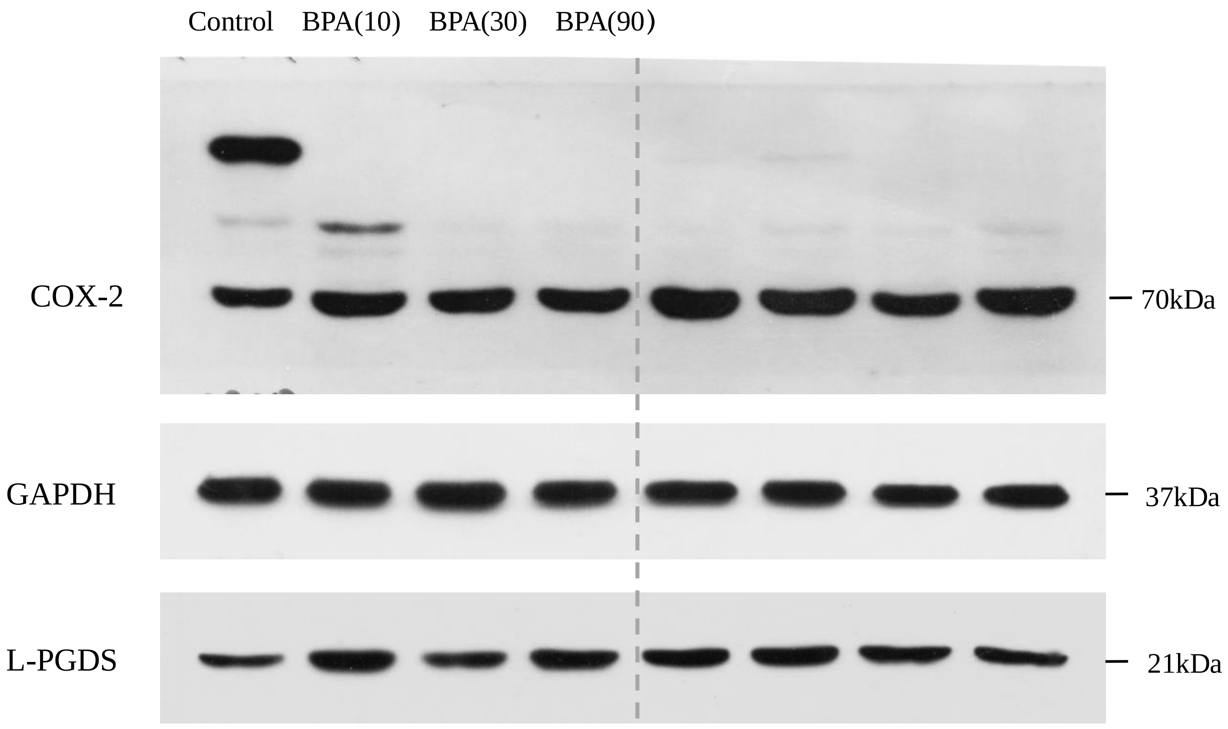

Figure 1. Original gel of Fig 7d in manuscript.

*Correspondence should be addressed to Jianhui Wu, Researcher, Tel: +86-21-64048232, E-mail: wujh_731@163.com.

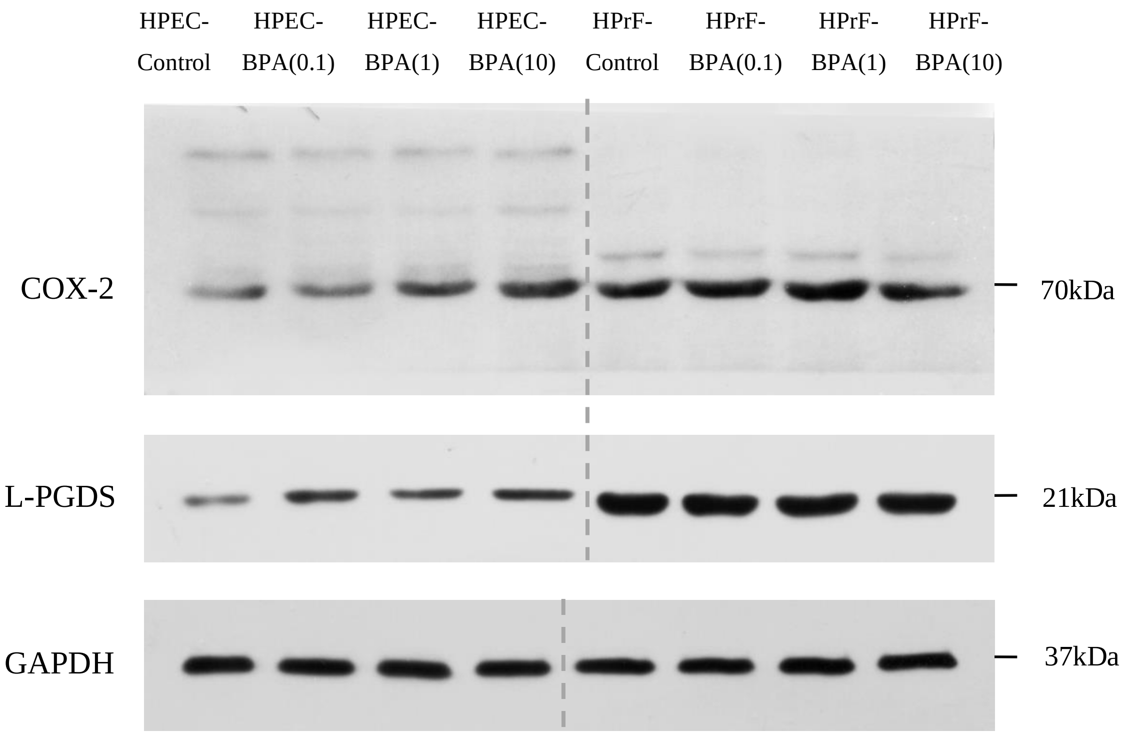

Figure 2. Original gel of Fig 9b,9d in manuscript.

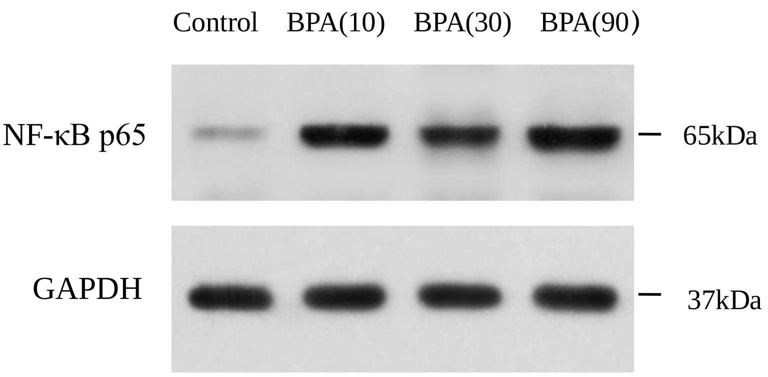

Figure 3. Original gel of Fig 8b in manuscript.
